# Clinical Characteristics, Risk Factors, and Outcomes of Patients with Polymicrobial *Klebsiella pneumoniae* Bloodstream Infections

**DOI:** 10.1155/2021/6619911

**Published:** 2021-06-19

**Authors:** Feizhen Song, Kai Zhang, Jianjiang Huang, Zhenhua Qian, Hongwei Zhou, Jiachang Cai, Cheng Zheng, Feifei Zhou, Wei Cui, Gensheng Zhang

**Affiliations:** ^1^Department of Critical Care Medicine, Second Affiliated Hospital, Zhejiang University School of Medicine, Hangzhou, Zhejiang 310009, China; ^2^Department of Critical Care Medicine, Shengzhou People's Hospital, Shaoxing, Zhejiang 312000, China; ^3^Department of Critical Care Medicine, Shaoxing Central Hospital, Shaoxing, Zhejiang 312000, China; ^4^Clinical Microbiology Laboratory, Second Affiliated Hospital, Zhejiang University School of Medicine, Hangzhou, Zhejiang 310009, China; ^5^Department of Critical Care Medicine, Taizhou Municipal Hospital, Taizhou, Zhejiang 318000, China; ^6^Department of Critical Care Medicine, Ningbo Medical Center, Li Huili Hospital, Ningbo, Zhejiang 315040, China

## Abstract

**Background:**

Polymicrobial *Klebsiella pneumoniae* bloodstream infection (KP-BSI) has been reported to account for more than 10% of all KP-BSI, but few studies have characterized polymicrobial KP-BSI. Our study investigated the clinical characteristics, risk factors, and outcomes of polymicrobial KP-BSI by comparing with monomicrobial KP-BSI.

**Methods:**

We conducted a single-center retrospective cohort study of patients with KP-BSI from 1 January 2013 to 31 December 2018 and collected the clinical data by reviewing electronic medical records.

**Results:**

Of the 818 patients with KP-BSI recruited, 13.9% (114/818) were polymicrobial KP-BSI. The severity of illness in polymicrobial and monomicrobial KP-BSI was similar, while the rate of resistance to carbapenems was obviously higher in polymicrobial KP-BSI (78.1% vs. 65.6%, *p* = 0.009). On multivariate analysis, hospitalization in burn ward (odds ratio (OR) 6.13, 95% confidence interval (CI) 2.00-18.76, *p* = 0.001) and intensive care unit (OR 2.39, 95% CI 1.05-5.43, *p* = 0.038) was independently associated with polymicrobial KP-BSI. Gram-negative bacteria accounted for the highest proportion (68.9%) among copathogens of polymicrobial KP-BSI, whereas gram-positive bacteria (22.9%) and *Candida* (8.2%) ranked the second and the third, respectively, with *Acinetobacter baumannii* being the most common (23.0%). Patients with polymicrobial KP-BSI had longer hospital days after BSI onset and total hospital days than patients with monomicrobial KP-BSI (median (interquartile range (IQR)), 19 (5, 39) vs. 12 (6, 25), 37 (21, 67) vs. 29 (16, 53), respectively, *p* < 0.05). The mortality did not differ between polymicrobial KP-BSI and monomicrobial KP-BSI (all *p* > 0.05).

**Conclusions:**

It was observed that polymicrobial KP-BSI accounted for a significant proportion among all KP-BSI in the current study. Hospitalization in burn ward and intensive care unit was an independent risk factor for the development of polymicrobial KP-BSI. The patients with polymicrobial KP-BSI had a higher rate of carbapenem-resistant *K. pneumoniae* and might have poor outcomes compared to monomicrobial KP-BSI.

## 1. Introduction

Bloodstream infections (BSIs) have high morbidity and mortality worldwide, with a crude in-hospital mortality rate of 12% to 20% [1–3]. *Klebsiella pneumoniae* is one of the most common pathogens in BSIs [4]. It is showed that *K. pneumoniae* BSIs (KP-BSIs) account for 6.6% to 9.9% among all BSIs and the mortality rate of KP-BSIs varies from 20% to 37% [4–6]. Moreover, with the widespread use of cephalosporins, extended-spectrum *β*-lactamase- (ESBL-) producing *K. pneumoniae* has become prevalent, resulting in limited selection of antibiotics and increased use of carbapenems [7, 8]. Therefore, carbapenem resistance among *K. pneumoniae* rises continually in the last decade [9, 10]. The prevalence of carbapenem-resistant KP-BSIs promotes mortality and length of hospital stay [11, 12]. Thus, KP-BSIs have posed a great public threat to healthcare.

Polymicrobial BSI occurs in 10.9% to 20.6% of patients with BSI and is associated with adverse outcomes [13–15]. Maria et al. have found that patients with polymicrobial BSI are characterized by higher Acute Physiology and Chronic Health Evaluation (APACHE) II score, more proportions of severe sepsis or septic shock, and higher mortality compared to patients with monomicrobial BSI. Previous studies focus on the clinical characteristics and outcomes of monomicrobial KP-BSI or all KP-BSI; few studies specifically investigate the prevalence and feature of polymicrobial KP-BSI among all KP-BSI [11, 16, 17]. Recently, Liu et al. [18] have reported that longer hospital length of stay and more prior use of carbapenems are risk factors of polymicrobial infection, but polymicrobial BSI involving *K. pneumoniae* will not increase the mortality of these patients. However, this study simply focused on KP-BSI in patients with complicated intra-abdominal infections rather than all KP-BSI patients and the sample size in the study was relatively small (only 98 patients). These results suggest that clinical features and outcomes of polymicrobial KP-BSI are still largely unknown.

To further address this issue, we herein performed the retrospective cohort study to investigate and analyze the differences of clinical characteristics, risk factors, and outcomes between polymicrobial KP-BSI and monomicrobial KP-BSI, attempting to improve the knowledge of polymicrobial KP-BSI by physicians.

## 2. Materials and Methods

### 2.1. Patients and Study Design

We conducted this retrospective cohort study in the Second Affiliated Hospital, Zhejiang University School of Medicine, which is a tertiary-level healthcare facility with 3200 beds in Hangzhou, China. The study was approved by the Ethics Committee of the Second Affiliated Hospital, Zhejiang University School of Medicine (NO.2019-119). This was a retrospective study, in which there were no interventions for the patients and their privacy and personal information were protected, so the written informed consent was waived.

Consecutive patients with *K. pneumoniae* positive in blood cultures during hospitalization were identified between 1 January 2013 and 31 December 2018. The relevant clinical data were obtained from the electronic medical records. Our analysis only included the first onset of monomicrobial or polymicrobial KP-BSI in each patient. Exclusion criteria were patients with age < 18 years old, without clinical manifestation or with data incomplete or missing.

### 2.2. Data Collection

The demographic data including age and gender, prior hospital days, prior healthcare interventions (during the 30 days before BSI onset), hospitalization ward at BSI onset, immunosuppression, sources of infection, healthcare-associated infection or not, and prior antimicrobial therapy were collected. Comorbid conditions were determined by surveying comorbidities and using the Charlson comorbidity index (CCI) [19]. Furthermore, the biological indicators including blood routine test, liver function, serum creatinine, procalcitonin, and C-reactive protein at the onset of BSI were recorded. APACHE II score, Sequential Organ Failure Assessment (SOFA) score, and Pitt bacteremia score were collected to assess the severity of the disease when BSI occurred [20–22]. Septic shock at the onset of BSI was assessed by the criteria of Sepsis-3 [23]. Appropriate empirical antimicrobial therapies were judged according to antimicrobial susceptibility testing results. Clinical outcomes were assessed by hospital days after BSI onset, total hospital days, and total intensive care unit (ICU) days. Additionally, 7-day, 14-day, 28-day, and in-hospital mortality was also evaluated.

### 2.3. Microbiological Analysis

Blood specimens were sent to the microbiology laboratory and cultured by the BacT/ALERT 3D system (Becton-Dickinson, Sparks, MD, USA). Bruker Daltonics Data Analysis was used to identify microbial species. Antimicrobial susceptibility testing was identified by the Vitek 2 automated system (bioMerieux, France) or the Kirby-Bauer Disk Diffusion method (Oxoid, UK). Minimum inhibitory concentrations (MICs) and inhibition zone diameters were interpreted according to the guidelines of the Clinical and Laboratory Standards Institute (CLSI) [24]. An MIC for ertapenem ≥ 1 mg/L or for imipenem or meropenem ≥ 2 mg/L was regarded as carbapenem resistant [24]. But tigecycline susceptibilities were judged by the U.S. Food and Drug Administration (FDA) break points, while colistin susceptibilities were determined by the European Committee on Antimicrobial Susceptibility Testing (EU-CAST) criteria [25, 26].

### 2.4. Definitions

KP-BSI referred to the isolation of *K. pneumoniae* in blood cultures accompanied by clinical manifestations of infection. If *K. pneumoniae* and other microorganisms were simultaneously isolated from the same set of blood cultures, it was regarded as polymicrobial KP-BSI [13]. BSI onset was considered to be the collection date of the first positive blood culture. If common commensal organisms, such as coagulase-negative staphylococci (CNS), viridans group streptococci, *Bacillus* spp., and *Corynebacterium* spp., were cultured from two or more blood specimens collected on separate occasions, they were considered to be pathogens [27]. Healthcare-associated BSI was defined as BSI occurring more than 48 hours after hospital admission or within 48 hours of a previous hospital discharge according to the criteria of the Centers for Disease Control and Prevention [27]. Immunosuppression included chemotherapy or radiotherapy within 30 days prior to culture, solid-organ transplantation or hematopoietic stem cell transplantation within 30 days prior to culture, and corticosteroid therapy with prednisone equivalent at a daily dose of ≥25 mg for more than 1 month or a cumulative dose of >700 mg within 3 months prior to culture [28]. Prior antimicrobial therapy referred to any antibiotic use for more than 48 hours in the past 30 days before BSI onset. Empirical antimicrobial therapy was regarded as appropriate if one or more antibiotics were in vitro sensitive against isolated pathogen or pathogens within 24 hours after BSI onset [29].

### 2.5. Statistical Analysis

The SPSS Version 18.0 was used for statistical analysis (IBM Corporation, Armonk, NY, USA). Continuous variables of normal distribution were presented as mean ± standard deviation (SD) and compared by Student's *t*-test, and continuous variables of nonnormal distribution were presented as median (interquartile range (IQR)) and compared by Mann–Whitney *U* test. Categorical variables were expressed as percentages and analyzed by Pearson *χ*^2^ test or Fisher's exact test as appropriate. Age was dichotomized at 65 years. Dichotomized age and other variables with *p* < 0.10 in the univariate analysis were used to perform the multivariate analysis by using binary logistic regression to estimate independent risk factors for polymicrobial KP-BSI. Odds ratios (ORs) and their 95% confidence intervals (CIs) were calculated. Survival analysis of polymicrobial or monomicrobial KP-BSI was made by using the Kaplan-Meier product limit method and compared by log-rank and Wilcoxon tests. All statistics tests were 2-tailed and considered statistically significant if *p* < 0.05.

## 3. Results

### 3.1. Demographic and Clinical Characteristics

**Figure 1:**
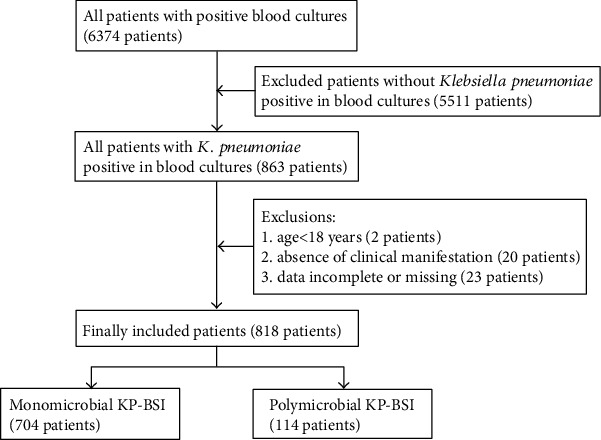
Flowchart of study participant enrollment.

In the six-year study period, 863 patients with at least one positive blood culture for *K. pneumoniae* were identified among 6374 patients with positive blood cultures. Two patients aged <18 years, 20 patients without clinical manifestation, and 23 patients with data incomplete or missing were excluded. Finally, we recruited 818 patients, among whom 114 (13.9%) patients were polymicrobial KP-BSI and 704 (86.1%) patients were monomicrobial KP-BSI (Figure 1).

The demographic data of enrolled patients are summarized in Table 1. The mean age of these patients with KP-BSI was 59.6 ± 15.8 years, with 39.5% (323/818) of them over 65 years old, and 71.4% (584/818) were male. The average age of patients with polymicrobial KP-BSI was lower than that of patients with monomicrobial KP-BSI (55.3 ± 18.1 vs. 60.3 ± 15.3 years, *p* = 0.006), but there was no substantially difference in gender. Trauma was the most prevalent comorbidity in polymicrobial KP-BSI, but without significant difference with monomicrobial KP-BSI (20.2% vs. 13.8%, *p* = 0.073). Compared to monomicrobial KP-BSI, polymicrobial KP-BSI had a lower score of CCI (media (IQR), 2 (1, 2) vs. 3 (2, 4), *p* = 0.011), less prevalence of solid tumor (12.3% vs. 22.7%, *p* = 0.011), more proportions of mechanical ventilation (74.6% vs. 58.2%), indwelling catheterization (87.7% vs. 70.2%), central venous catheterization (88.6% vs. 69.9%), parenteral nutrition (41.2% vs. 25.6%), and antimicrobial therapy (91.2% vs. 79.4%) during the 30 days prior to BSI onset (all *p* < 0.05). With regard to hospitalization ward at BSI onset, the occurrence of polymicrobial KP-BSI was the highest in the ICU (64.9%), followed by burn wards (20.2%) and general wards (20.2%), which was different from that of monomicrobial KP-BSI. Polymicrobial KP-BSI was more frequently caused by skin and soft tissue infection (17.5% vs. 4.4%, *p* < 0.001). In addition, polymicrobial KP-BSI had a higher proportion of healthcare-associated infection than monomicrobial KP-BSI (94.7% vs. 87.4%, *p* = 0.023).

### 3.2. Biological Indicators and Severity of Illness

The biological indicators and severity of illness of the two groups are showed in Table 2. Patients with polymicrobial KP-BSI had higher level of glutamic-oxaloacetic transaminase (GOT) than patients with monomicrobial KP-BSI (*p* < 0.05). However, other indicators of liver function, serum creatinine, blood routine test, procalcitonin, and C-reactive protein showed no significant differences. Besides, the incidence of septic shock, SOFA score, APACHE II score, and the proportion of Pitt bacteremia score > 4 points when BSI occurred were similar.

### 3.3. Risk Factors for Polymicrobial KP-BSI

Univariate analyses of risk factors for polymicrobial KP-BSI identified significant association with prior mechanical ventilation, prior indwelling urinary catheter, prior central venous catheter, prior parenteral nutrition, prior antimicrobial therapy, hospitalization in burn ward or ICU at BSI onset, skin and soft tissue infection, and healthcare-associated infection (Table 3). In the multivariate analysis, independent risk factors for polymicrobial KP-BSI development contained hospitalization in burn ward (OR 6.13, 95% CI 2.00-18.76, *p* = 0.001) and ICU (OR 2.39, 95% CI 1.05-5.43, *p* = 0.038) at BSI onset (Table 3). By contrast, age ≥ 65 years (OR 0.53, 95% CI 0.29-0.98, *p* = 0.042) was identified as a low risk factor for polymicrobial KP-BSI.

### 3.4. Isolated Microorganisms from Patients with Polymicrobial KP-BSI

**Figure 2:**
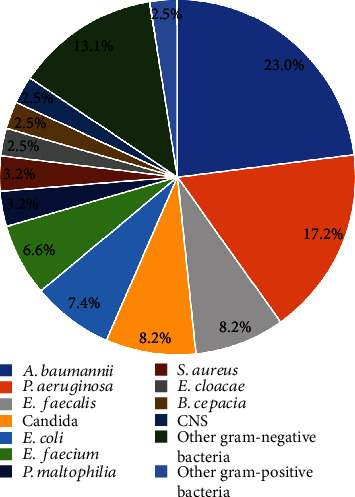
Microorganisms isolated from 114 patients with polymicrobial KP-BSI. Abbreviations: *A. baumannii*: *Acinetobacter baumannii*; *P. aeruginosa*: *Pseudomonas aeruginosa*; *E. faecalis*: *Enterococcus faecalis*; *E. coli*: *Escherichia coli*; *E. faecium*: *Enterococcus faecium*; *P. maltophilia*: *Pseudomonas maltophilia*; *S. aureus*: *Staphylococcus aureus*; *E. cloacae*: *Enterobacter cloacae*; *B. cepacia*: *Burkholderia cepacia*; CNS: coagulase-negative staphylococci.

We identified 122 microorganisms other than *K. pneumoniae* from 114 patients with polymicrobial KP-BSI. Of note, 107 patients (93.8%) had one microorganism, 6 patients (5.3%) had two microorganisms, and only 1 patient (0.9%) had three organisms. Gram-negative bacteria, gram-positive bacteria, and *Candida* accounted for 68.9%, 22.9%, and 8.2% of the 122 pathogens isolated, respectively. The most common microorganism was *Acinetobacter baumannii* (23.0%, 28/122), followed by *Pseudomonas aeruginosa* (17.2%, 21/122) and *Enterococcus faecalis* and *Candida* (both 8.2%, 10/122) (Figure 2).

### 3.5. Antimicrobial Resistance and Antimicrobial Therapy

Details of antimicrobial resistance of *K. pneumoniae* and antimicrobial therapy are shown in Table 4. The resistance of *K. pneumoniae* to amoxicillin-clavulanic acid, ceftriaxone, cefoperazone-sulbactam, and piperacillin-tazobactam occurred more frequently in polymicrobial KP-BSI than in monomicrobial KP-BSI (all *p* < 0.05). The rate of resistance to carbapenems in polymicrobial KP-BSI was significantly higher than in monomicrobial KP-BSI (78.1% vs. 65.6%, *p* = 0.009). The total rate of appropriate empiric antimicrobial therapy in the KP-BSI was less than 50%, whereas there was no difference between the two groups.

### 3.6. Outcomes

**Figure 3:**
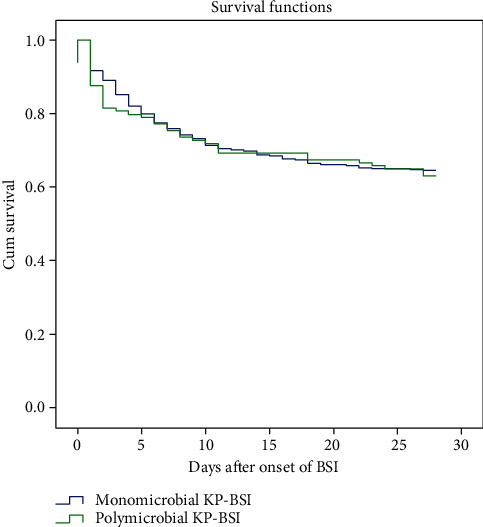
Kaplan-Meier estimates of survival in patients with polymicrobial KP-BSI and monomicrobial KP-BSI.

By comparing polymicrobial KP-BSI to monomicrobial KP-BSI, it was found that the former had longer hospital days after BSI onset and total hospital days than the latter (median (IQR), 19 (5-39) vs. 12 (6-25), 37 (21-67) vs. 29 (16-53), respectively, *p* < 0.05). The length of total ICU stay in the polymicrobial group was slightly longer than that in the monomicrobial group (14 (IQR, 0-33) vs. 9 (IQR, 0-26)), though no statistical difference was observed (*p* = 0.219) (Table 5). The 7-day, 14-day, 28-day, and in-hospital mortality in polymicrobial KP-BSI patients did not differ from that in monomicrobial KP-BSI patients (all *p* > 0.05) (Table 5). Survival curve analysis also showed that the 28-day survival of the two groups was similar (Figure 3).

## 4. Discussion

This is the current largest study about the epidemiology of polymicrobial KP-BSI in mainland, China. Our study had several main findings as follows. (1) Polymicrobial KP-BSI was not a rare phenomenon, which accounted for 13.9% of total KP-BSI. (2) Many factors like prior mechanical ventilation, prior indwelling urinary catheter, and prior parenteral nutrition were associated with the occurrence of polymicrobial KP-BSI (Table 3). Moreover, hospitalizations in burn ward and ICU were independent risk factors. (3) Gram-negative bacteria accounted for the highest proportion among copathogens of polymicrobial KP-BSI, followed by gram-positive bacteria and *Candida*, and the most common microorganism was *A. baumannii*. (4) The outcomes of patients with polymicrobial KP-BSI might be poor, including longer hospital days after BSI onset and prolonged lengths of hospital stay.

Our current study found that the proportion of polymicrobial KP-BSI was 13.9% among total KP-BSI, which was consistent with previous reports that ranged from 11.7% to 21.0% [15, 16, 30]. In Zheng et al.'s study, the proportion of polymicrobial enterococcal BSI was even higher [31]. Therefore, polymicrobial BSI is no longer a rare phenomenon. A recent prospective study showed that about 10.9% of patients with BSI suffered from polymicrobial BSI and *K. pneumoniae* ranked the second most common accompanying microorganism following *Escherichia coli* [15].

In the present study, we noted that prior healthcare interventions including mechanical ventilation, indwelling urinary catheter, central venous catheter, and parenteral nutrition, prior antimicrobial therapy, skin and soft tissue infection, and healthcare-associated infection were risk factors of polymicrobial KP-BSI. In a matched case-control study, the presence of malignancy and a history of hospitalization within 90 days were regarded as risk factors of polymicrobial BSI [32]. However, this study did not specifically refer to the polymicrobial BSI of a certain microorganism. In a study of polymicrobial KP-BSI, the use of carbapenems after admission and prior BSI were risk factors of polymicrobial KP-BSI, but parenteral nutrition and mechanical ventilation were not associated with polymicrobial KP-BSI [18]. Different study populations and different factors may account for the different findings between the study mentioned above and our study. Linder et al. [33] showed that patients with skin and soft tissue infection might be caused by a variety of pathogens including *Klebsiella* species and were susceptible to polymicrobial infections, which accounted for 26.8% of the cases. Curiously, there was no difference in the proportion of central line-associated infection between monomicrobial KP-BSI and polymicrobial KP-BSI. This might be because the catheter tip culture cannot be performed without the removal of central venous catheter so that not all central line-associated infections were identified. From the current study, we also learned that hospitalization in burn ward and ICU was independently associated with polymicrobial KP-BSI. Of the 53 patients in our burn wards, only one was admitted not because of burns but because of diabetic foot infection, and 23 of them had polymicrobial KP-BSI. Tang et al. [34] suggested that *K. pneumoniae* was the second common organism causing BSI and a high proportion (51.1%) of polymicrobial infection was observed in the burn patients. Rose et al. [14] found that over one-fifth of patients with bacteremia in ICU were polymicrobial, which points to high prevalence of polymicrobial BSI in ICU. But age ≥ 65 years was identified as a low risk factor for polymicrobial KP-BSI and the CCI of the patients with polymicrobial KP-BSI was lower, perhaps because patients with trauma and patient hospitalization in burn ward accounted for more than one-third of all patients with polymicrobial KP-BSI in this study, and these patients were younger and had fewer comorbidities than other patients.

Like in Liu et al.'s study [18], gram-negative bacteria accounted for the highest proportion among copathogens of polymicrobial KP-BSI in our study. But the most common copathogen was *A. baumannii* in our study, while it was *E. coli* in Liu et al.'s study. Liu et al.'s study included only patients with complicated intra-abdominal infections, of which *E. coli* was also the most prevalent pathogenic bacterium [35]. In the present study, patients with KP-BSI from various sources of infection were included and patients in the ICU accounted for 64.9% of the total patients with polymicrobial KP-BSI. The EUROBACT study pointed out that gram-negative bacteria were the leading cause of BSI in the ICU, with *Acinetobacter* spp. being the most frequent causative agent [36]. Besides, it was reported that the most common source of *A. baumannii* was lower respiratory tract infection, followed by primary BSI and catheter-related infection [37], whereas in this study, lower respiratory tract and central-line associated source of polymicrobial KP-BSI accounted for approximately 50.0% (Table 1). Thus, these might partially explain gram-negative bacteria especially *A. baumannii* account for a high proportion of copathogens among polymicrobial KP-BSI.

The outcomes of patients with polymicrobial KP-BSI might be poor, including longer hospital days after BSI onset and prolonged lengths of hospital stay. A higher rate of carbapenem-resistant *K. pneumoniae* and a higher proportion of burn in polymicrobial KP-BSI might ascribe to the poor outcomes compared with monomicrobial KP-BSI. Consistent with our findings, a longer length of hospital stay was also observed in patients with carbapenem-resistant KP-BSI than those with carbapenem-susceptible KP-BSI in Tian et al.'s study [12]. Nevertheless, differences in mortality were not significant either in our study or in other studies for polymicrobial BSI of other pathogens such as *Candida*, *Enterococcus*, *Staphylococcus aureus*, or *A. baumannii* [31, 37–39]. Kohler et al.'s meta-analysis indicated that appropriateness of empirical antimicrobial therapy was an important contributor to the observed difference in mortality between patients with carbapenem-resistant KP-BSI and patients with carbapenem-susceptible KP-BSI [40]. In our study, although polymicrobial KP-BSI had a significantly higher rate of carbapenem-resistant *K. pneumoniae* (CRKP) than monomicrobial KP-BSI, their rates of appropriate empirical antimicrobial therapy were similar. Other variables associated with mortality included markers of severity of the underlying disease [40]. In the present study, except for GOT, other biological indicators such as glutamic-pyruvic transaminase (GPT), serum creatinine, white blood count, procalcitonin, and C-reactive protein were similar between polymicrobial KP-BSI and monomicrobial KP-BSI. Pitt bacteremia score in polymicrobial KP-BSI was higher than that in monomicrobial KP-BSI. As far as we know, Pitt bacteremia score greater than 4 points can be considered as critically ill and predict an increased mortality in patients with BSI [41, 42]. Therefore, we compared the ratio of Pitt score above 4 points between the two groups, but found no difference (Table 2). Additionally, the incidence of septic shock, SOFA score, and APACHE II score were equally high among patients with polymicrobial KP-BSI and monomicrobial KP-BSI. These indicated that there was no difference in the severity of the disease between polymicrobial KP-BSI and monomicrobial KP-BSI. Besides, younger age, fewer solid tumors, and similar lower respiratory tract focus of infection might also contribute to the similar mortality [17].

We realize that there are some limitations in this study. Due to the retrospective nature of this study, some sources of bloodstream infections were hard to be identified and might be mistakenly classified. Additionally, due to the reliance on documentation in the electronic medical records, some important information could not be obtained accurately, which led to information bias. Finally, this was a single-center study, and the results might not be extended to other centers and settings. Therefore, further multicenter, prospective studies are needed to explore the clinical features of polymicrobial KP-BSI.

## 5. Conclusions

Polymicrobial KP-BSI accounted for a significant proportion among all KP-BSI. Hospitalization in burn ward and ICU was an independent risk factor for the development of polymicrobial KP-BSI. Polymicrobial KP-BSI had no influence on the mortality, but was related to longer hospital days after BSI onset and total hospital days. In addition, the rate of CRKP in polymicrobial KP-BSI was significantly higher. Thus, we should pay more attentions to polymicrobial KP-BSI.

## Figures and Tables

**Table 1:** Demographic and clinical characteristics of patients with monomicrobial and polymicrobial *Klebsiella pneumoniae* bloodstream infection.

Characteristics	Total (*n* = 818)	Monomicrobial (*n* = 704)	Polymicrobial (*n* = 114)	*p* value
Age (years), mean (±SD)	59.6 ± 15.8	60.3 ± 15.3	55.3 ± 18.1	0.006
Age ≥ 65 years	323 (39.5%)	290 (41.2%)	33 (28.9%)	0.013
Male sex, *n* (%)	584 (71.4%)	508 (72.2%)	76 (66.7%)	0.229
Comorbidity, *n* (%)				
Diabetes mellitus	143 (17.5%)	122 (17.3%)	21 (18.4%)	0.776
Solid tumor	174 (21.3%)	160 (22.7%)	14 (12.3%)	0.011
Hematological malignancy	35 (4.3%)	32 (4.5%)	3 (2.6%)	0.459
COPD	30 (3.7%)	26 (3.7%)	4 (3.5%)	1.000
Chronic heart failure	48 (5.9%)	41 (5.8%)	7 (6.1%)	0.894
Chronic renal failure	15 (1.8%)	14 (2.0%)	1 (0.9%)	0.708
Chronic liver disease	32 (3.9%)	28 (4.0%)	4 (3.5%)	1.000
Cerebrovascular disease	145 (17.7%)	128 (18.2%)	17 (14.9%)	0.396
Trauma	120 (14.7%)	97 (13.8%)	23 (20.2%)	0.073
CCI, median (IQR)	3 (1, 4)	3 (2, 4)	2 (1, 2)	0.011
Immunosuppression, *n* (%)	109 (13.3%)	95 (13.5%)	14 (12.3%)	0.724
Prior hospital days, median (IQR)	13 (6, 26)	13 (5, 26)	14 (9, 25)	0.112
Prior healthcare interventions^∗^, *n* (%)				
Mechanical ventilation	495 (60.5%)	410 (58.2%)	85 (74.6%)	0.001
Indwelling urinary catheter	594 (72.6%)	494 (70.2%)	100 (87.7%)	<0.001
Central venous catheter	593 (72.5%)	492 (69.9%)	101 (88.6%)	<0.001
CRRT	92 (11.2%)	75 (10.7%)	17 (14.9%)	0.182
Surgery	448 (54.8%)	376 (53.4%)	72 (63.2%)	0.052
Parenteral nutrition	227 (22.7%)	180 (25.6%)	47 (41.2%)	0.001
Blood transfusion	123 (15.0%)	100 (14.2%)	23 (20.2%)	0.098
Prior antimicrobial therapy^∗^, *n* (%)	703 (85.9%)	599 (79.4%)	104 (91.2%)	0.003
Hospitalization ward at BSI onset, *n* (%)				<0.001
General	303 (37.0%)	286 (40.6%)	17 (14.9%)	
Burn	53 (6.5%)	30 (4.3%)	23 (20.2%)	
ICU	462 (56.5%)	388 (55.1%)	74 (64.9%)	
Primary site of infection, *n* (%)				
Lower respiratory tract	174 (21.3%)	149 (21.2%)	25 (22.0%)	0.853
Urinary tract	50 (6.1%)	47 (6.7%)	3 (2.6%)	0.094
Central line associated	133 (16.3%)	108 (15.3%)	25 (22.0%)	0.077
Intra-abdomen	62 (7.6%)	55 (7.8%)	7 (6.1%)	0.531
Biliary tract	71 (8.7%)	64 (9.1%)	7 (6.1%)	0.299
Liver abscess	38 (4.6%)	37 (5.3%)	1 (0.9%)	0.069
Skin and soft tissue	51 (6.2%)	31 (4.4%)	20 (17.5%)	<0.001
Others^∗∗^	74 (9.0%)	67 (9.5%)	7 (6.1%)	0.244
Unknown	165 (20.2%)	146 (20.7%)	19 (16.7%)	0.315
Healthcare-associated infection, *n* (%)	723 (88.4%)	615 (87.4%)	108 (94.7%)	0.023

SD: standard deviation; IQR: interquartile range; COPD: chronic obstructive pulmonary disease; CCI: Charlson comorbidity index; CRRT: continuous renal replacement therapy; BSI: bloodstream infection; ICU: intensive care unit. ^∗^During the 30 days before BSI onset. ^∗∗^Central nervous system infection, bone and joint infection, mediastinal infection, endocarditis, etc.

**Table 2:** Biological indicators and severity of illness of patients with monomicrobial and polymicrobial *Klebsiella pneumoniae* bloodstream infection.

Indicator	Total (*n* = 818)	Monomicrobial (*n* = 704)	Polymicrobial (*n* = 114)	*p* value
Blood routine test, median (IQR)				
WBC (×10^9^/L)	9.6 (5.9, 15.0)	9.7 (5.9, 15.2)	8.8 (5.2, 14.0)	0.254
ANC (×10^9^/L)	8.5 (5.0, 13.4)	8.6 (5.0, 13.6)	7.9 (4.1, 12.5)	0.267
PLT (×10^9^/L)	137 (68, 214)	137 (68, 215)	128 (56, 204)	0.560
Liver function, median (IQR)				
Albumin (g/L)^∗^	31.5 (27.3, 35.6)	31.5 (27.6, 35.6)	29.5 (26.3, 35.0)	0.112
GPT (U/L)^∗^	39 (24, 69)	39 (24, 68)	41 (25, 74)	0.416
GOT (U/L)^∗^	41 (25, 75)	41 (25, 73)	45 (31, 85)	0.047
Total bilirubin (*μ*mol/L)	20.1 (12.1, 40.8)	19.8 (11.9, 40.4)	21.4 (12.6, 45.6)	0.522
Scr (*μ*mol/L), median (IQR)	64 (46, 96)	64 (46, 97)	62 (42, 91)	0.398
PCT (ng/mL), median (IQR)^∗∗^	1.75 (0.51, 8.46)	1.63 (0.47, 8.29)	2.09 (0.62, 10.11)	0.394
CRP (mg/L), median (IQR)^∗∗∗^	102.1 (62.2, 191.4)	103.7 (62.5, 190.1)	92.4 (60.4, 201.3)	0.566
Sepsis shock, *n* (%)	258 (31.5%)	217 (30.8%)	41 (36.0%)	0.273
SOFA score, median (IQR)	5 (3, 8)	5 (3, 8)	6 (3, 10)	0.082
APACHE II score, median (IQR)	15 (11, 21)	15 (11, 21)	16 (11, 21)	0.340
Pitt bacteremia score, median (IQR)	3 (1, 5)	3 (1, 5)	4 (2, 6)	0.005
Pitt bacteremia score > 4 points, *n* (%)	275 (33.6%)	229 (32.5%)	46 (38.3%)	0.101

IQR: interquartile range; WBC: white blood count; ANC: absolute neutrophil count; PLT: platelet; GPT: glutamic-pyruvic transaminase; GOT: glutamic-oxaloacetic transaminase; Scr: serum creatinine; PCT: procalcitonin; CRP: C-reactive protein; SOFA: Sequential Organ Failure Assessment; APACHE: Acute Physiology and Chronic Health Evaluation. ^∗^7 missing values in the monomicrobial group and 1 missing value in the polymicrobial group. ^∗∗^121 missing values in the monomicrobial group and 16 missing values in the polymicrobial group. ^∗∗∗^54 missing values in the monomicrobial group and 10 missing values in the polymicrobial group.

**Table 3:** Risk factors for polymicrobial *Klebsiella pneumoniae* bloodstream infection by univariate and multivariate analyses.

Variable	Univariate		Multivariate	
	OR (95% CI)	*p* value	OR (95% CI)	*p* value
Age ≥ 65 years	0.58 (0.38-0.90)	0.014	0.53 (0.29-0.98)	0.042
Solid tumor	0.48 (0.26-0.86)	0.013	0.81 (0.41-1.60)	0.539
Trauma	1.58 (0.95-2.62)	0.075	1.53 (0.85-2.76)	0.154
CCI	0.89 (0.81-0.98)	0.022	1.11 (0.97-1.28)	0.141
Prior mechanical ventilation	2.10 (1.34-3.29)	0.001	0.99 (0.49-2.00)	0.975
Prior indwelling urinary catheter	3.04 (1.70-5.43)	<0.001	1.29 (0.54-3.06)	0.571
Prior central venous catheter	3.35 (1.84-6.10)	<0.001	1.63 (0.73-3.61)	0.233
Prior surgery	1.50 (0.99-2.25)	0.053	0.70 (0.43-1.15)	0.159
Prior parenteral nutrition	2.04 (1.36-3.08)	0.001	1.455 (0.96-2.51)	0.076
Prior blood transfusion	1.53 (0.92-2.53)	0.100	1.00 (0.57-1.76)	0.987
Prior antimicrobial therapy	2.70 (1.38-5.29)	0.004	0.90 (0.36-2.21)	0.815
Hospitalization ward at BSI onset				
General ward	1 (reference)		1 (reference)	
Burn ward	12.90 (6.21-26.79)	<0.001	6.13 (2.00-18.76)	0.001
ICU	3.21 (1.85-5.56)	<0.001	2.39 (1.05-5.43)	0.038
Urinary tract infection	0.38 (0.12-1.24)	0.107	0.59 (0.17-2.04)	0.400
Central line-associated infection	1.55 (0.95-2.53)	0.079	0.90 (0.36-2.21)	0.412
Liver abscess	0.16 (0.02-1.17)	0.072	0.38 (0.05-3.17)	0.372
Skin and soft tissue infection	4.62 (2.53-8.43)	<0.001	1.91 (0.77-4.78)	0.164
Healthcare-associated infection	2.60 (1.11-6.10)	0.028	0.68 (0.22-2.12)	0.508

OR: odds ratio; CI: confidence interval; CCI: Charlson comorbidity index; ICU: intensive care unit.

**Table 4:** Antimicrobial resistance of *Klebsiella pneumoniae* and antimicrobial therapy in patients with monomicrobial and polymicrobial *K. pneumoniae* bloodstream infection.

Bacteriology	Total (*n* = 818)	Monomicrobial (*n* = 704)	Polymicrobial (*n* = 114)	*p* value
Antimicrobial resistance				
Cefoxitin (650 vs. 109)^∗^	514 (67.2%)	432 (66.5%)	82 (75.2%)	0.070
Ceftazidime (601 vs. 94)^∗^	483 (69.5%)	411 (68.4%)	72 (76.6%)	0.108
Ceftriaxone (659 vs. 112)^∗^	552 (71.6%)	460 (69.8%)	92 (82.1%)	0.007
Cefepime (704 vs. 114)^∗^	531 (64.9%)	448 (63.6%)	83 (72.8%)	0.057
Amoxicillin-clavulanic acid (653 vs. 112)^∗^	541 (70.7%)	448 (68.6%)	93 (89.0%)	0.002
Piperacillin-tazobactam (704 vs. 114)^∗^	551 (67.4%)	463 (65.8%)	88 (77.2%)	0.016
Cefoperazone-sulbactam (654 vs. 103)^∗^	515 (68.0%)	436 (66.7%)	79 (76.7%)	0.042
Amikacin (704 vs. 114)^∗^	357 (43.6%)	303 (43.0%)	54 (47.4%)	0.387
Ciprofloxacin (704 vs. 114)^∗^	515 (63.0%)	438 (62.2%)	77 (67.5%)	0.274
Levofloxacin (704 vs. 114)^∗^	498 (60.9%)	423 (60.1%)	75 (65.8%)	0.247
Carbapenems (704 vs. 114)^∗^	551 (67.4%)	462 (65.6%)	89 (78.1%)	0.009
Tigecycline (605 vs. 94)^∗^	184 (26.3%)	158 (26.1%)	26 (27.7%)	0.752
Colistin (247 vs. 37)^∗^	5 (1.8%)	3 (1.2%)	2 (5.4%)	0.128
Appropriate empirical antimicrobial therapy, *n* (%)	387 (47.3%)	341 (48.4%)	46 (40.4%)	0.109

^∗^The figures in parentheses were the total numbers of *K. pneumoniae* used for antimicrobial susceptibility testing in both groups.

**Table 5:** Outcomes of patients with monomicrobial and polymicrobial *Klebsiella pneumoniae* bloodstream infection.

Parameter	Total (*n* = 818)	Monomicrobial (*n* = 704)	Polymicrobial (*n* = 114)	*p* value
Hospital days after BSI onset, median (IQR)	12 (6, 27)	12 (6, 25)	19 (5, 39)	0.028
Total hospital days, median (IQR)	30 (17, 55)	29 (16, 53)	37 (21, 67)	0.006
Total ICU days, median (IQR)	9 (0, 28)	9 (0, 26)	14 (0, 33)	0.219
7-day mortality, *n* (%)	198 (24.2%)	170 (24.1%)	28 (24.6%)	0.924
14-day mortality, *n* (%)	255 (31.2%)	220 (31.3%)	35 (30.7%)	0.907
28-day mortality, *n* (%)	292 (35.7%)	250 (35.1%)	42 (36.8%)	0.783
In-hospital mortality, *n* (%)	332 (40.1%)	283 (40.2%)	49 (43.0%)	0.574

IQR: interquartile range; ICU: intensive care unit.

## Data Availability

The data used and/or analyzed in this study are available from the corresponding author on reasonable request.
